# COMPASS‐ND: A unique cohort across the dementia spectrum

**DOI:** 10.1002/alz70855_100224

**Published:** 2025-12-23

**Authors:** Michael J Borrie, Natalie A. Phillips, Sarah Best, Panayiota Anastasiou‐Ventura, Turaç Aydoğan, Camille Beaudoin, Jonathan Beuk, Isabella Celotto, Lauren Cole, Samir Das, Jennifer Fogarty, Celine Fouquet, Logane Gnassi, Charlie Henri‐Bellemare, Randi Pilon, Jenna Sands, Ana Stirbu, Julia Truemner, Senny Chan, Afra Tucker, Julianna Gajraj, Jaspreet Bhangu, Howard Chertkow

**Affiliations:** ^1^ Division of Geriatric Medicine, Western University, London, ON, Canada; ^2^ Lawson Research Institute, London, ON, Canada; ^3^ St. Joseph's Health Care London, London, ON, Canada; ^4^ Parkwood Institute, London, ON, Canada; ^5^ Schulich School of Medicine and Dentistry, Western University, London, ON, Canada; ^6^ Concordia University, Montréal, QC, Canada; ^7^ Lady Davis Research Institute, Montréal, QC, Canada; ^8^ Lady Davis Institute, Montréal, QC, Canada; ^9^ McGill Centre for Integrative Neuroscience, Montréal, QC, Canada; ^10^ Baycrest Health Sciences, Toronto, ON, Canada; ^11^ McGill Centre for Integrative Neuroscience, Montréal, QC, Canada; ^12^ Institut universitaire de gériatrie de Montréal, Montréal, QC, Canada; ^13^ Lady Davis Resesarch Institute, Montréal, QC, Canada; ^14^ Lady Davis Research Insitute, Montréal, QC, Canada; ^15^ University of Toronto, Toronto, ON, Canada; ^16^ Western University, London, ON, Canada; ^17^ Lady Davis Institute for Medical Research, Jewish General Hospital, Montreal, QC, Canada; ^18^ Kimel Family Centre for Brain Health and Wellness and Anne & Allan Bank Centre for Clinical Research Trials, Baycrest Health Sciences, Toronto, ON, Canada; ^19^ Division of Neurology, Department of Medicine, University of Toronto, Toronto, ON, Canada; ^20^ Baycrest and Rotman Research Institute, Toronto, ON, Canada; ^21^ McGill University, Montreal, QC, Canada

## Abstract

**Background:**

The Comprehensive Assessment of Neurodegeneration and Dementia (COMPASS‐ND) used wide inclusion criteria recruiting participants across the spectrum from normal cognition to cognitive impairment to all types of clinical dementia, including mixed dementia. 1173 participants comprising 11 diagnostic cohorts, completed screening, clinical, neuropsychology, sensory and motor assessments, magnetic resonance imaging (MRI) and provided biomarker bio‐samples.

**Method:**

Recruitment was from advertising and specialty clinics for neurocognitive disorders and/or memory impairment. Robust data monitoring and cleaning occurred on the baseline and Time 2 data. Genetic, MRI, Cerebrospinal Fluid (CSF) and the majority of blood biomarkers analyses have been completed.

**Result:**

Data releases uploaded to the Longitudinal Online Research and Imaging System (LORIS) include alpha numeric baseline data, neuroimaging analyses, CSF, genetic and blood biomarkers. 40 autopsies have been completed.

119 data access requests have been submitted to the Data Access Subcommittee.

All returning participants will repeat Time 3 extensive clinical and neuropsychology assessments, MRI and provide bio‐samples.

Phase III new recruitment will include 400 diverse participants, with up to Grade 12 education, who are not cognitively impaired, or who have cognitive impairment, but not dementia. The balance of each cohort for sex/gender will be a consideration.

**Conclusion:**

COMPASS‐ND fills a knowledge gap by recruiting participants representing the full spectrum of neurodegeneration. This distinguishes COMPASS‐ND from other large‐scale studies. Challenges and strategies for engagement and research site level funding will be discussed.

Data are available to registered researchers and trainees in Canada and around the world through data access requests.

